# The Biology and Immature Stages of the Moss-Eating Flea Beetle *Cangshanaltica* *fuanensis* sp. nov. (Coleoptera, Chrysomelidae, Galerucinae, Alticini), with Description of a Fan-Driven High-Power Berlese Funnel

**DOI:** 10.3390/insects11090571

**Published:** 2020-08-26

**Authors:** Yongying Ruan, Alexander S. Konstantinov, Albert F. Damaška

**Affiliations:** 1 School of Applied Chemistry and Biological Technology, Shenzhen Polytechnic, Shenzhen 518055, China; 2 Systematic Entomology Laboratory, USDA, Smithsonian Institution, National Museum of Natural History, P.O. Box 37012, Washington, DC 20013-7012, USA; alex.konstantinov@ars.usda.gov; 3 Department of Zoology, Faculty of Science, Charles University, Viničná 7, 128 00 Prague, Czech Republic; albert.damaska@natur.cuni.cz

**Keywords:** Berlese extraction, Berlese-Tullgren funnel, biodiversity, bryobiont, China, larva, moss inhabiting

## Abstract

**Simple Summary:**

The immature stages and the biology of the moss inhabiting flea beetles are poorly understood. In this study, a new species of moss-eating flea beetles—*Cangshanaltica fuanensis* sp. nov. is described; the morphology of the adult and immature stages is described and illustrated. The life history and remarkable biological features of this species are revealed. Females deposit one large egg at a time; egg length equals 0.4–0.5 times the female body length. Females lay and hide each egg under a spoon-shaped moss leaf. There are only two ovarioles on each side of the ovary in the female reproductive system, which has not been reported before in Chrysomelidae. Besides, a modified fan-driven Berlese funnel is designed for faster extraction of moss inhabiting flea beetles. We suggest this improved device could also be useful for collecting other ground-dwelling arthropods.

**Abstract:**

The biology of the moss and leaf litter inhabiting flea beetles is poorly understood. In this study, a new species of moss-eating flea beetles *Cangshanaltica fuanensis* sp. nov. is described; the morphology of adult and immature stages is examined and illustrated. Its life history and biology are studied. The remarkable and unique biological features are revealed: (1) females deposit one large egg at a time, egg length equals 0.4–0.5 times the female body length, these are unusual in Chrysomelidae; (2) females have only two ovarioles on each side of the ovary, which has not been reported in other Chrysomelidae species; (3) females lay and hide each egg under a spoon-shaped moss leaf; (4) cannibalism of a second instar larva on an egg was observed. Both adults and larvae feed on moss and are polyphagous; their feces mainly consist of un-digested moss fragments; high humidity is essential for the survival of eggs and larvae and expedites the hatching. In addition, a modified fan-driven Berlese funnel is designed for faster extraction of moss inhabiting flea beetles. This device could also be used for collecting other ground-dwelling arthropods. Its working diagram is illustrated and described.

## 1. Introduction

The moss and leaf litter inhabiting flea beetles (Alticini) are not well studied due to their relatively cryptic living environment. Their tremendous diversity has been highlighted by recent studies (e.g., [1,2,3,4,5,6,7,8,9,10,11]). Most known moss inhabiting leaf beetles belong to the tribe Alticini [12]. Recently, an unusual Galerucini species was discovered feeding on and inhabiting moss cushions [12]. In New Zealand, several genera in the subfamily Chrysomelinae (i.e., *Nanomela, Zeaphilon, Aphilon* and *Maurodus*) are associated with mosses and liverworts [13]. These studies suggest that other Chrysomelidae groups could also inhabit and feed on moss. It also means our current knowledge of the diversity of the moss inhabiting leaf beetles is still insufficient.

The moss-eating genus *Cangshanaltica* was described from Yunnan, China by Konstantinov et al. [2]; subsequently, four more species were described from Thailand, China (Hongkong) and Philippines [3,7,9]. The wide distribution and the small number of described species suggested a hidden diversity in this genus.

Three species of moss inhabiting flea beetle larvae were already described by previous studies: *Ivalia korakundah* Prathapan, Konstantinov and Duckett [14]; *Mniophila muscorum* (Koch) [15]; *Distigmoptera borealis* Blake [10]. Despite these studies, the biology and life history of moss inhabiting flea beetles are not well understood. In this study, we describe a new species of *Cangshanaltica*, with data on its biology, life history, and morphology of the immature stages.

Prior to this study, traditional Berlese funnels were frequently used to collect moss inhabiting flea beetles (for methods see [11]). These devices usually use light bulbs (40–150 W) as heating elements. Processing moss with moderate-to-high moisture using the light-bulb-driven funnels is very time consuming (usually more than 12 h for a standard size load). This process often yields only a few specimens considering that flea beetles are usually scarce in the moss, especially in daylight. In 2012, we used the traditional light-bulb-driven Berlese funnels to process moist moss cushions in Shuyang, Fuan, Fujian Province, China. Despite our endeavors for several days, only one individual of *Cangshanaltica fuanensis* sp. nov. was extracted. In 2014, an attempt to collect more specimens for the taxonomical study had failed as this species has a very low population density. To process more moss and therefore to obtain more individuals within a limited time, a modified Berlese funnel applying a high-power heating element and a fan was developed and tested. In 2019, using the modified fan-driven Berlese funnel, we extracted numerous living larvae and adults of *Cangshanaltica fuanensis* sp. nov. and several other flea beetle species that dwell in the moss cushions. The adults of *C. fuanensis* sp. nov. were then transferred to rearing containers, and their biology was observed. The modified fan-driven Berlese funnel was also tested and proven suitable for collecting other ground-dwelling arthropods. A working diagram of the device is illustrated and described here.

## 2. Materials and Methods

### 2.1. Morphological Methods

Observations of the habitus and diagnostic characters of flea beetles were made using the Nikon SMZ645 stereomicroscope and Nikon OPTIPHOT microscope. The genitalia with the last few tergites was torn off using sharp insect pins attached to plastic sticks. The tissues surrounding the aedeagus were cleared. Female genitalia and accompanied structures (the last tergites) were immersed into hot 10% NaOH solution for 30 s (or the appropriate time required to soften irrelevant tissue). The extra tissues surrounding the genitalia were carefully removed using insect pins. For photography, the female genitalia were mounted on slides with glycerin; male genitalia were glued to paper points. Digital images were taken with Canon D800 camera attached to Canon MP-E 65 mm Lens or a modified lens (consists of a Nikon M Plan lens, a manual aperture unit, and an adjustable extension tube).

Flea beetles are treated as “Alticini” in this study.

Adult morphological terminology follows Konstantinov et al. (2013) [2]. The terminology of larval tubercles follows Kimoto (1962) [16] and Takizawa (2005) [17]. Abbreviations of tubercles: ***D***, *dorsal*; ***DL***, *dorsolateral*; ***EP***, *epipleural*; ***ES***, *eusternal*; ***P***, *pleural*; ***PS***, *parasternal*; ***SS***, *sternellar*. Position of tubercles denoted by lowercase letter: ***a***, *anterior*; ***p***, *posterior*; ***i***, *interior*; ***e***, *exterior.*

Abbreviations for insect collections. **ADPC**: personal collection of A. Damaška. **IZCAS**: Institute of Zoology, Chinese Academy of Sciences, Beijing, China. **SZPT**: School of Applied Chemistry and Biological Technology, Shenzhen Polytechnic, Shenzhen, Guangdong, China.

Mosses were identified using “Flora Bryophytorum Sinicorum” [18]. A bryophyte taxonomist was consulted.

### 2.2. Biological Methods

The activities of *Cangshanaltica fuanensis* sp. nov. are hard to observe in nature; most of the biological data were collected based on lab-reared individuals. A rearing container was designed for rearing *C. fuanensis* sp. nov. Transparent plastic containers (15 cm × 7 cm × 5 cm) were selected and placed in a north-facing room to avoid direct sunlight. Two rectangle openings were carved in the sides of the container; they were sealed by non-woven fabrics using adhesive, allowing for air circulating and preventing other organisms from coming into the container. A thick layer of moist paper towel was placed at the bottom of the container in order to maintain proper humidity and avoid larvae from drowning in water drops; a thin layer of soil was placed above the paper towel in order to provide nutrition for host plant and site for larvae to pupate; fresh host plant moss was collected and placed loosely above the soil layer. Distilled water was sprayed on the moss once a day to maintain humidity using a small spraying device.

Adults extracted from moss were transferred to the rearing container (ca. 25 °C, 90–100% air humidity were maintained). Approximately 30 parent (F_0_) adults were collected from wild and raised until they were dead. Their first filial generation (F_1_) was also maintained alive in the same container for the remainder of their lives. An F_2_ generation was not successfully bred. The biology and behavior of numerous individuals of different stages were observed (ca. 30 F_0_ adults, 35 F_1_ eggs, 30 F_1_ larvae, 10 F_1_ pupae, and 6 F_1_ adults).

The genitalia of the adults of F_1_ were dissected to confirm that they were conspecific with their parent generation.

### 2.3. DNA Barcoding

Specimens used for the DNA barcoding were treated as follows: DNA was extracted by Qiagen DNEasy Blood and Tissue kit or GenAid Genomic DNA Mini kit in a thermo-shaker, and 50 μL of elution buffer was used in the final step. In every specimen, the body was perforated by breaking abdominal tergites for better DNA yield during the extraction. After DNA extraction, the voucher specimens were mounted on cards and housed in ADPC (see Type Material section). For PCR reactions, we used a modified protocol with a commercially prepared premix (PPP Mix with MgCl_2_ added, Top-Bio Czech Republic). We used *cox1* barcoding primers: forward LCO1490 (5′-GGTCAACAAATCATAAAGATATTGG-3′) and reverse HCO2198 (5′-TAAACTTCAGGGTGACCAAAAAATCA-3′) [19]. PCR was performed in a 13 μL total volume of the mixture, containing 6.25 μL of PPP Mix, 4.75 μL of PCR ddH_2_O, 1.0 μL of each primer and 1.0 μL of the DNA extract. The following PCR program was used: 94 °C for 180 s + 35 × (94 °C for 30 s, 48 °C for 45 s, 72 °C for 60 s) + 72 °C for 480 s. PCR products were purified by adding 0.5 μL Exonuclease 1 (Exo1 (20 U/μL)) (ThermoFisherScientific) and 1.0 μL Thermosensitive Alkaline Phosphatase (FastAP (1 U/μL)) (ThermoFisherScientific); the mixture was incubated in a thermocycler for 37 °C for 15 min and 80 °C for 15 min. Samples were sequenced by using Sanger sequencing. Raw sequence data were edited by using Geneious 9.1.7 software (Biomatters). Sequences were submitted to GenBank, accession numbers: MT891188.

### 2.4. The Modified Fan-Driven Berlese Funnel

The majority of the specimens used in this study were extracted from moss using the modified Berlese funnel. A portable fan heater was used as the heating source, 500–1800 W, switchable for high-power and low-power modes depends on the moisture contained in the samples; it mainly consists of a PTC (positive temperature coefficient) ceramic heating elements, and a fan installed above it. The fan heater was hung 20 cm above the funnel using a tripod or other supporters; it generated and propelled the warm airflow down to the samples (soil, moss, or leaf litter) placed in the funnel. By this means, the warm airflow brought away the moisture, dried up the samples and drove the arthropods down to the beaker placed under the funnel. One or two pieces of plastic mesh screens were placed at the bottom of the funnel; soil, moss, or leaf litter was loosely placed above the mesh screen. A large cylindrical plastic beaker was placed on the ground and attached under the funnel in order to collect arthropod specimens and keep the funnel from toppling. The gap between funnel and beaker was sealed by tape; a layer of thick moist paper towel was placed in the beaker to keep organisms alive (paper towel could be replaced with ethanol to kill specimens). The moist paper towel was critical for keeping specimens alive: in the earlier stage of the extraction process, it provided humidity for the survival of organisms (especially insect larvae); while in a later stage, it absorbed the condensed water aggregated at the bottom of the beaker and prevented organisms from drowning.

The extracting process required approximately 2–4 h for lightly moist moss (moss volume = 6–8 L, loosely placed in the funnel); around 4–8 h for entirely wet moss (moss volume = 6–8 L, loosely placed in the funnel); around 4–5 h for moist and fluffy humus soil (volume = 4–5 L, loosely placed in the funnel).

## 3. Results

### 3.1. Taxonomy

**Genus *Cangshanaltica* Konstantinov et al. 2013.**

*Cangshanaltica* Konstantinov, Chamorro, Prathapan, Ge and Yang, 2013: 6.

**Type species.***Cangshanaltica nigra* Konstantinov, Chamorro, Prathapan, Ge and Yang, 2013: 16, by original designation.

**Distribution.** China (Yunnan, Hongkong, Fujian); Thailand (Chiang Mai), Philippines (Luzon, Mindanao).

**Figure 1:**
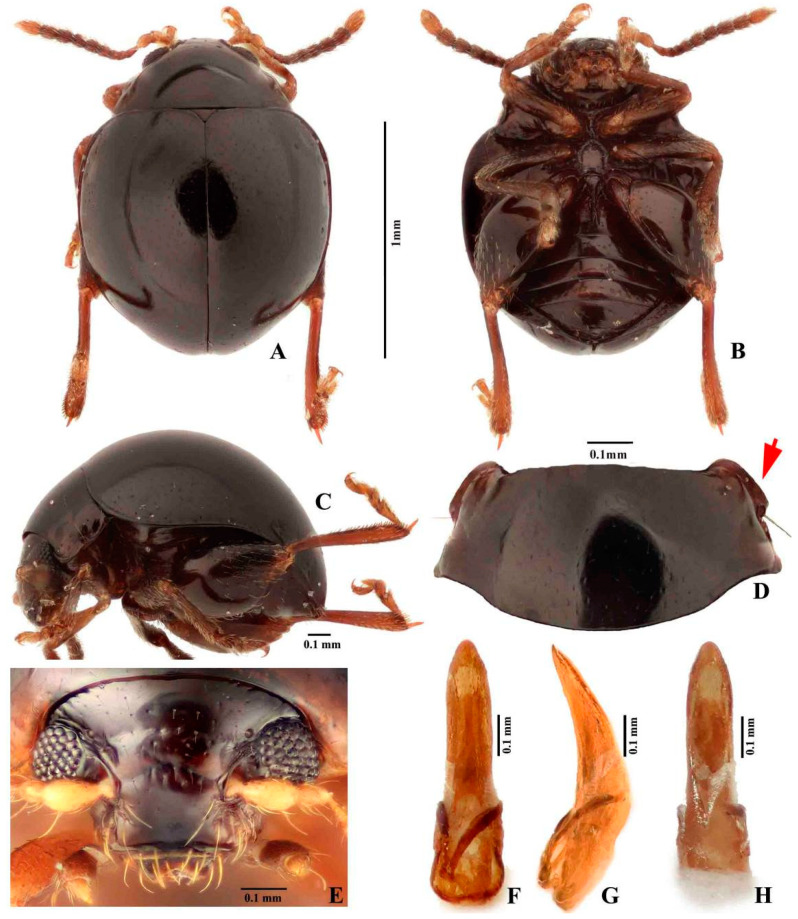
**Adult morphology of *Cangshanaltica fuanensis* sp. nov.** (**A**): Holotype, dorsal view. (**B**): Holotype, ventral view. (**C**): Holotype, lateral view. (**D**): Pronotum, holotype. (**E**): Head, paratype. (**F**): Aedeagus of holotype, ventral view. (**G**): Aedeagus of holotype, lateral view. (**H**): Aedeagus of holotype, dorsal view.

**Figure 2:**
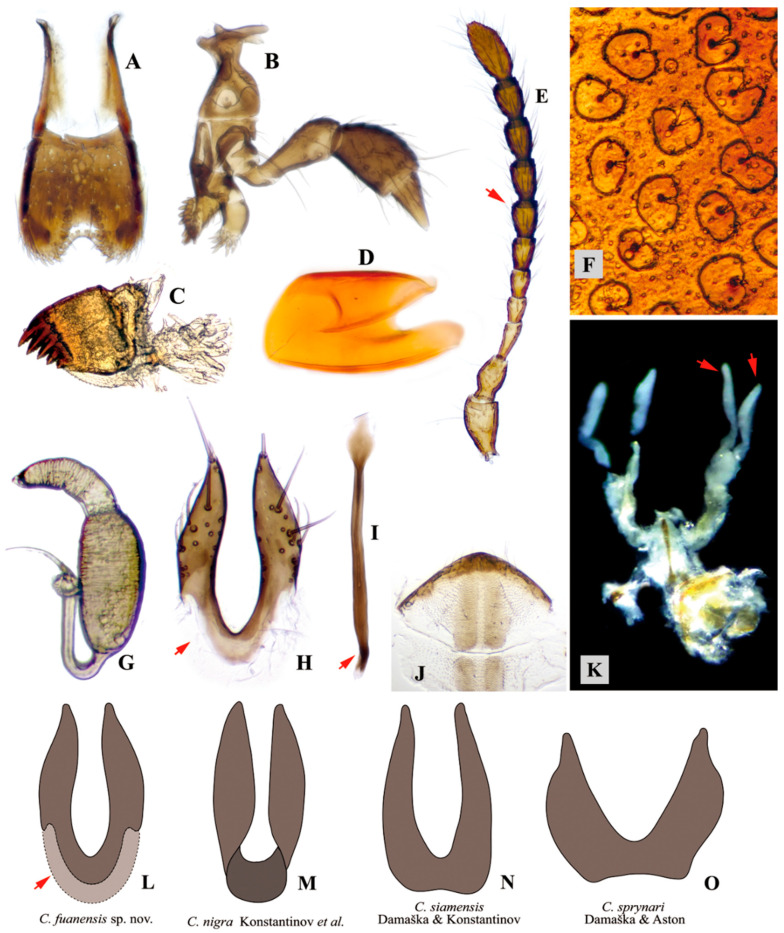
**Adult morphology of genus *Cangshanaltica*.** (**A**–**L**), *Cangshanaltica fuanensis* sp. nov.; (**M**–**O**), other *Cangshanaltica* species. (**A**)**:** Labrum. (**B**)**:** Maxilla. (**C**): Mandible. (**D**): Metafemoral spring of hind leg. (**E**): Antenna, indicating the apical part of the 7th antennomere only very slightly produced. (**F**): Elytra under high magnification (immersed in glycerin, showing kidney-shaped elytral punctures and small granules. (**G**): Spermatheca. (**H**): Vaginal palpi; the arrow indicates the unsclerotized area. (**I**): Tignum. (**J**): Female pygidium. (**K**): Female reproductive system, only two ovarioles present on each side of ovary; the arrows indicate the two ovarioles on the right ovary. (**L**–**O**): Vaginal palpi of *Cangshanaltica* species, showing interspecific variation, insets M–O are redrawn based on [2,3,7].

***Cangshanaltica fuanensis* Ruan, Konstantinov and Damaška, new species** (Figure 1 and Figure 2)

**Type locality.** Shuyang, Fuan, Fujian Prov., China.

**Distribution.** China (Fujian).

**Etymology.** This species is named after the type locality—Fuan city. Specific epithet is a noun in apposition.

**Diagnosis.** This new species is assigned to the genus *Cangshanaltica* based on its pronotal anterolateral setiferous pore situated at the middle of the lateral margin of pronotum, and a longitudinal ridge on the first abdominal ventrite is strongly developed, extending to near posterior margin. It may be distinguished from other known species of *Cangshanaltica* by the shape of the aedeagus and female genitalia, particularly the distinct U-shaped unsclerotized area on the anterior part of the vaginal palpi (Figure 2L, indicated by arrow).

**Description of adult.** Male body length 1.35–1.55 mm, width 1.10–1.20 mm; female body length 1.50–1.70 mm, width 1.25–1.35 mm. Ratio of body length to body width: 1.20–1.30. Dorsum and head smooth, not reticulate, dark brown. Venter slightly lighter than dorsum: chestnut brown to dark brown. Antennae: antennomeres I–II yellow-brown, II–IV yellow-brown to chestnut brown, V–X chestnut brown, XI yellow-brown. Legs chestnut brown. Legs and antennae covered with yellow setae.

Head (Figure 1E). Head hypognathous. Vertex smooth and shiny, with small and sparse punctures bearing short setae. Antennal calli poorly delimited, smooth, triangular, with lighter color than vertex. Supracallinal sulcus absent, midfrontal and suprafrontal sulci barely visible, supraantennal and supraorbital sulci moderately deep and straight. Frontal ridge wide, slightly wider than width of eye. Labrum with three pairs of setae. Mandibles symmetrical, palmate; each mandible with five sharp teeth, mesal side with a membranous lobe bearing dense microtrichia. Antennomere VII with weak distal protrusion (almost invisible) (Figure 2E). Proportions of antennomere lengths: 100:70–72:57–59:38–39:44–46:52–54:59–61:61–63:61–63:61–63:119–121.

Thorax. Pronotum (Figure 1D) strongly convex, ratio of pronotum width (measured at posterior edge) to length: 2.0–2.2. Lateral margin with anterolateral setiferous pore situated nearly in midlength. Elytra strongly convex, humeral calli absent. Elytra with punctures confused, sparse, minute and shallow in dorsal view, kidney-shaped sculpture present when immersed in glycerin (Figure 2F). Hind wings absent. Procoxal cavity open externally. Dorsal side of metatibia with a row of minute spines situated between basal 1/3 and tibial apex. Length of metatibial apical spur to length of metatarsomere: 0.50–0.60. Proportions of metatarsomere lengths: 100: 6–37:44–45:70–72. First male protarsomere as large as that of female.

Abdomen. Longitudinal ridge on first abdominal ventrite strongly developed, extending to near posterior margin. Last visible abdominal ventrite of male has same shape as female.

Genitalia. Median lobe of aedeagus (Figure 1F–H) in ventral view: widest at middle or base; ventral surface with middle longitudinal area raised; sides narrowing from middle to apex; apex rounded, without denticle; widest near basal opening. Median lobe of aedeagus in lateral view: evenly curved ventrad, apex slightly bent dorsad, widest near middle.

Receptacle of spermatheca with external side convex, internal side almost straight to slightly convex (Figure 2G). Spermathecal pump much shorter and narrower than receptacle, slightly curved, apex without denticle. Tignum spear-shaped; posterior part short, oval to diamond-shaped, weakly sclerotized, much wider than anterior part; anterior part narrow and long, crooked near apex (Figure 2I, indicated by arrow). Vaginal palpi fused at base (anterior part), twice as long as wide, with base partly unsclerotized (Figure 2H, indicated by arrow); vaginal palpus: posterior half with lateral sides sinuate and gradually narrowed, posterior apex acute to narrowly rounded.

Variation. Adults with external characters almost invariable except for a slight variation of body size. Shape of median lobe of aedeagus: with middle part slightly dilated at middle part in ventral and lateral views in some cases; apex invariable, always slightly bending dorsally in lateral view. Shape of female genitalia: internal side of spermathecal receptacle slightly varied from slightly convex to almost straight; vaginal palpi invariable including the unsclerotized area; tignum invariable including the crooked anterior part (Figure 2I, indicated by arrow) near anterior apex.

**Barcode sequence**. The *cox1* barcode is accessible on GenBank, accession numbers: MT891188.

**Type Material** (information is recorded verbatim from labels). Holotype: ♂ (IZCAS), labels: (1) China, **Fujian Prov.**, Fuan, Shuyang, 250 m, 27.159° N 119.677° E, 27-IV-2019, sifted from moss. (2) HOLOTYPE *Cangshanaltica fuanensis* sp. nov. Des. Ruan et al. 2019.

Paratypes: 1♀ (IZCAS), labels: (1) **Fujian Prov.**, Fuan, Shuyang Village, 368 m, 27°09′32″ N, 119°40′34″ E, 2012.VIII.13, Host: Hypnaceae? Leg. Yongying R [label partly written in Chinese]; (2) PARATYPE *Cangshanaltica fuanensis* sp. nov. Des. Ruan et al. 2019. • 1♀ (IZCAS), labels: (1) China, **Fujian Prov.**, Fuan, Shuyang, 250 m, 27.159° N 119.677° E, 27-IV-2019, sifted from moss; (2) PARATYPE *Cangshanaltica fuanensis* sp. nov. Des. Ruan et al. 2019. • 2♂2♀ (IZCAS), labels: (1) China, **Fujian Prov.**, Fuan, Shuyang, 290 m, 27.1611° N 119.6763° E, 16-VIII-2019, Extracted from moss, Leg. Y. Ruan; (2) PARATYPE *Cangshanaltica fuanensis* sp. nov. Des. Ruan et al. 2019. • 1♂ (ADPC), labels: (1) China, **Fujian Prov.**, Fuan, Shuyang, 290 m, 27.1611° N 119.6763° E, 3-X-2019, Extracted from moss, Leg. Y. Ruan; (2) *Cangshanaltica fuanensis* des. Ruan, 2019; (3) PARATYPE *Cangshanaltica fuanensis* sp. nov. Des. Ruan et al. 2019; (4) VOUCHER SPECIMEN A. F. Damaška coll., AFD-265.• 1♂ (ADPC), labels: (1) China, **Fujian Prov.**, Fuan, Shuyang, 290 m, 27.1611° N 119.6763° E, 3-X-2019, Extracted from moss, Leg. Y. Ruan; (2) *Cangshanaltica fuanensis* des. Ruan, 2019; (3) PARATYPE *Cangshanaltica fuanensis* sp. nov. Des. Ruan et al. 2019. • 3♀5♂ (SZPT), labels: (1) China, **Fujian Prov.**, Fuan, Shuyang, 290 m, 27.1611° N 119.6763° E, 3-X-2019, Extracted from moss, Leg. Y. Ruan; (2) PARATYPE *Cangshanaltica fuanensis* sp. nov. Des. Ruan et al. 2019. • 3♂10♀ (SZPT), labels: (1) China, **Fujian Prov.**, Fuan, Shuyang, 290 m, 27.1611° N 119.6763° E, 13-II-2020, Extracted from moss, Leg. Y. Ruan; (2) PARATYPE *Cangshanaltica fuanensis* sp. nov. Des. Ruan et al. 2020.

**Host plants** (see “Biology” section for more information)**.** Adults feed on *Hypnum plumaeforme* (Hypnaceae) and *Racopilum* cf. *aristatum* (Racopilaceae) in nature, they also casually feed on *Bazzania tridens* (Lepidoziaceae), Hylocomiaceae sp. and Thuidiaceae sp. In a lab environment; moss spores were found in their intestinal tract. Larvae: feed on both *Hypnum plumaeforme* and *Racopilum* cf. *aristatum* in a lab environment.

**Remarks.** By the external habitus, *Cangshanaltica fuanensis* sp. nov. is similar to other non-metallic species of *Cangshanaltica* (*C. nigra* Konstantinov et al. [2]; *C. siamensis* Damaška and Konstantinov [3]; and *C. sprynari* Damaška and Aston [7]).

*Cangshanaltica fuanensis* sp. nov. may be distinguished from *C. nigra* by the following characters: dorsum dark brown throughout (in *C. nigra*, dorsum black with slight green-blue luster on elytra); protrusion at distal part of antennomere VII barely visible (in *C. nigra*, protrusion at distal part of antennomere VII strongly developed, clearly visible); in lateral view, apex of median lobe of aedeagus very slightly bent dorsally (in *C. nigra*, apex of median lobe of aedeagus bent ventrally).

*Cangshanaltica fuanensis* sp. nov. may be distinguished from *C. siamensis* by the following characters: body length 1.35–1.70 mm, width 1.10–1.35 mm (in *C. siamensis*, body length 1.94–2.21 mm, width 1.45–1.62 mm); middle-longitudinal ridge on first abdominal ventrite long and strongly developed, extending to near posterior margin (in *C. siamensis*, middle-longitudinal ridge on first abdominal ventrite short, weakly developed, not extending past midlength of first abdominal ventrite); in lateral view, apex of median lobe of aedeagus very slightly bent dorsally (in *C. siamensis*, apex of median lobe of aedeagus very slightly bent ventrally).

*Cangshanaltica fuanensis* sp. nov. may be distinguished from *C. sprynari* in by the following characters: surface of pronotum and elytra smooth, with fine punctures (in *C. sprynari*, dorsum with coarse punctures); middle-longitudinal ridge on first abdominal ventrite almost extending to posterior margin (in *C. sprynari*, middle-longitudinal ridge on first abdominal ventrite short, not extending to posterior margin); median lobe of aedeagus in ventral view, apical fourth of median lobe gradually narrowed, without an abruptly narrowing step (in *C. sprynari*, apical quarter of median lobe has an abruptly narrowing step); median lobe of aedeagus in lateral view, apex slightly bent dorsally (in *C. sprynari*, apex straight).

The main sexually dimorphic features present in other genera of flea beetles are absent in some *Cangshanaltica*, e.g., in *C. nigra*: male does not have larger first pro- and meso- tarsomeres; apex of last visible abdominal ventrite has the same shape in female and male [2]. This condition also occurs in *C. fuanensis* sp. nov.: females are almost indistinguishable from males by external characters, except for the slightly larger body size. However, in *C. fuanensis* sp. nov., the female tignum could be seen through abdominal ventrites when they are alive or preserved in ethanol (but hardly visible when the specimens are dry).

Although the four non-metallic species of *Cangshanaltica* are very similar to each other in external characters, the shape of genitalia is useful to separate species, especially the female vaginal palpi (see Figure 2L–O).

### 3.2. Morphology of Immature Stages

#### 3.2.1. Larval Morphology

**Figure 3:**
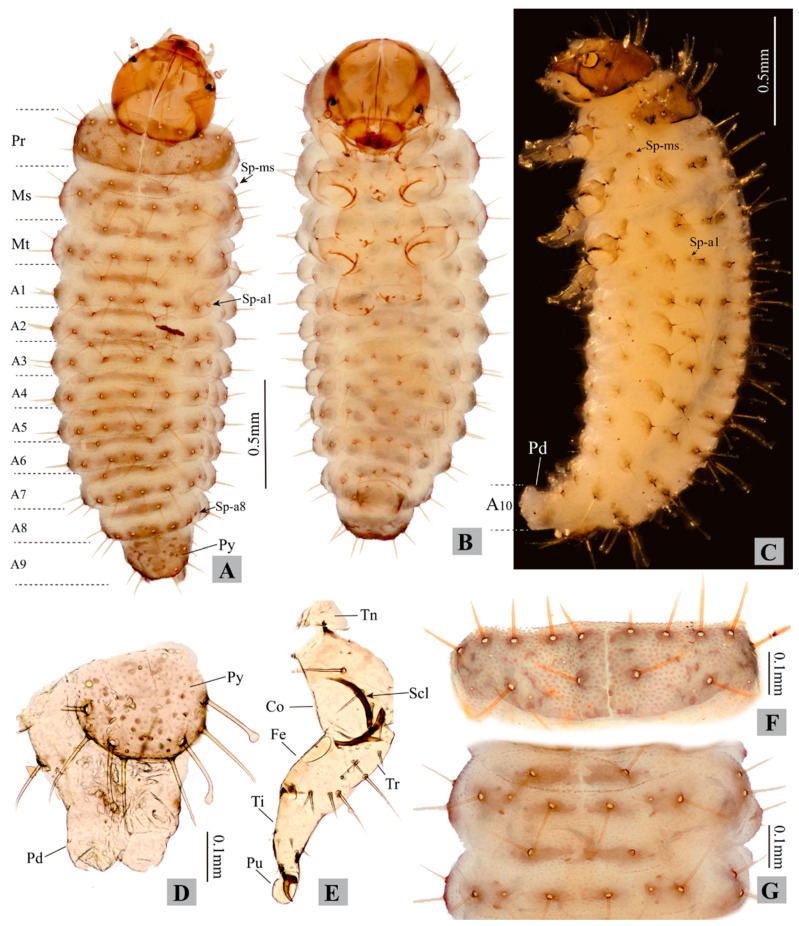
**Larval morphology of *Cangshanaltica fuanensis* sp. nov.** (specimens mounted in glycerin). (**A**): Habitus of 3rd instar, dorsal view. (**B**): Habitus of 3rd instar, ventral view. (**C**): Habitus of 3rd instar, lateral view. (**D**): Pygidium and pygopod. (**E**): Thoracic leg. (**F**): Pronotum. (**G**): Meso- and metanotum. (Abbreviations: **A1**–**A10**, *abdominal segments I*–*X*; ***Co***, *coxa*; ***Fe***, *femur*; ***Ms***, *mesothorax*; ***Mt***, *metathorax*; ***Pd***, *pygopod*; ***Pr***, *prothorax*; ***Pu***, *Pulvillus*; ***Py***, *pygidium*; ***Scl***, *sclerotization*; ***Sp***, *spiracle*; ***Sp-a1***, *1st abdominal spiracle*; ***Sp-a8***, *8th abdominal spiracle*; ***Sp-ms***, *mesothoracic spiracle*; ***Ti***, *Tibia* (*Tibia-tarsus*); ***Tn***, *trochantin*; ***Tr***, *trochanter*.)

**Figure 4:**
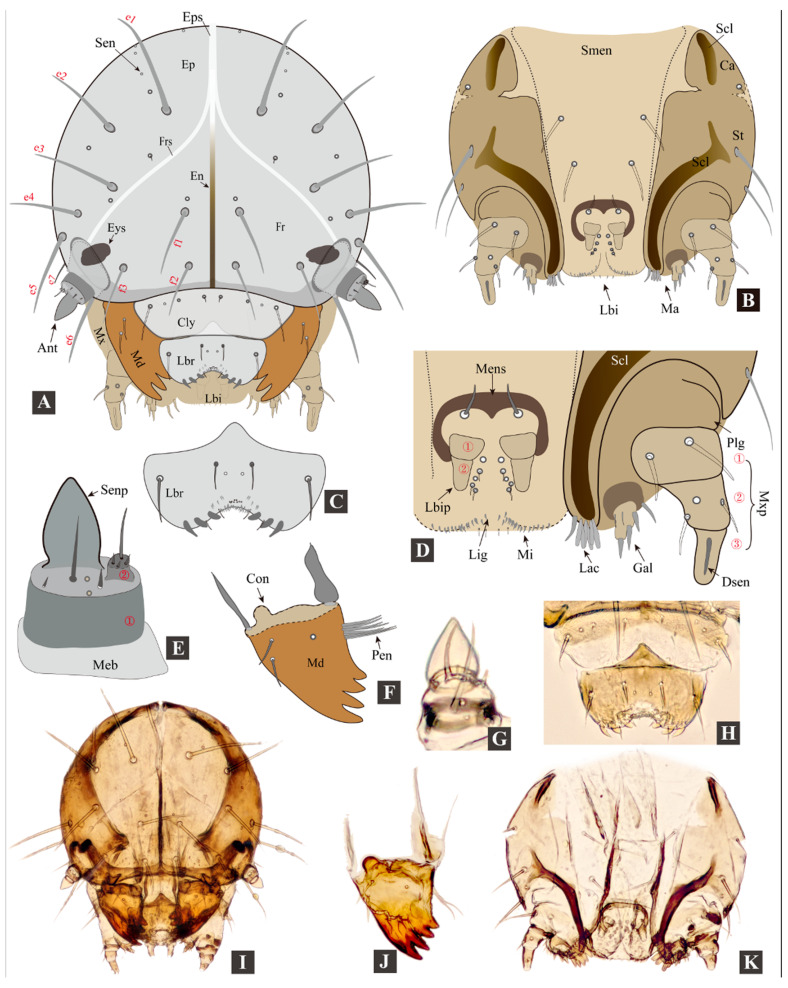
**Larval morphology of *Cangshanaltica fuanensis* sp. nov.** (**A**): Head; (**B**): maxilla and labium; (**C**): labrum; (**D**): apical part of labium and maxilla; (**E**): antenna; (**F**): mandible. (**G**): antenna; (**H**): labrum and clypeus; (**I**): head; (**J**): mandible; (**K**): maxilla and labium. (Abbreviations: ***Ant***, *antenna*; ***Ca***, *cardo*; ***Cly***, *clypeus*; ***Con***, *condyle*; ***Dsen**, digitiform sensillum*; ***e1***–***e7***, *epicranial setae*; ***En***, *endocarina*; ***Ep***, *epicranium*; ***Eps***, *epicranial suture*; ***Eys***, *eyespot*; ***f1***–***f3***, *frontal setae*; ***Fr***, *frons*; ***Frs***, *frontal suture*; ***Gal**, galea*; ***Lac***, *lacinia*; ***Lbi***, *labium*; ***Lbr***, *labrum*; ***Lbip***, *labial palpus*; ***Lig***, *ligula*; ***Ma***, *mala*; ***Md***, *mandible*; ***Meb***, *membrane*; ***Mens***, *mental sclerite*; ***Mi***, *microtrichia*; ***Mx***, *maxilla*; ***Mxp***, *maxillary palpus*; ***Pen***, *penicillus*; ***Plg***, *palpiger*; ***Scl***, *sclerotization*; ***Sen***, *sensillum*; ***Senp***, *sensory papilla*; ***Smen***, *submentum*; ***St***, *stipes*.).

**Figure 5:**
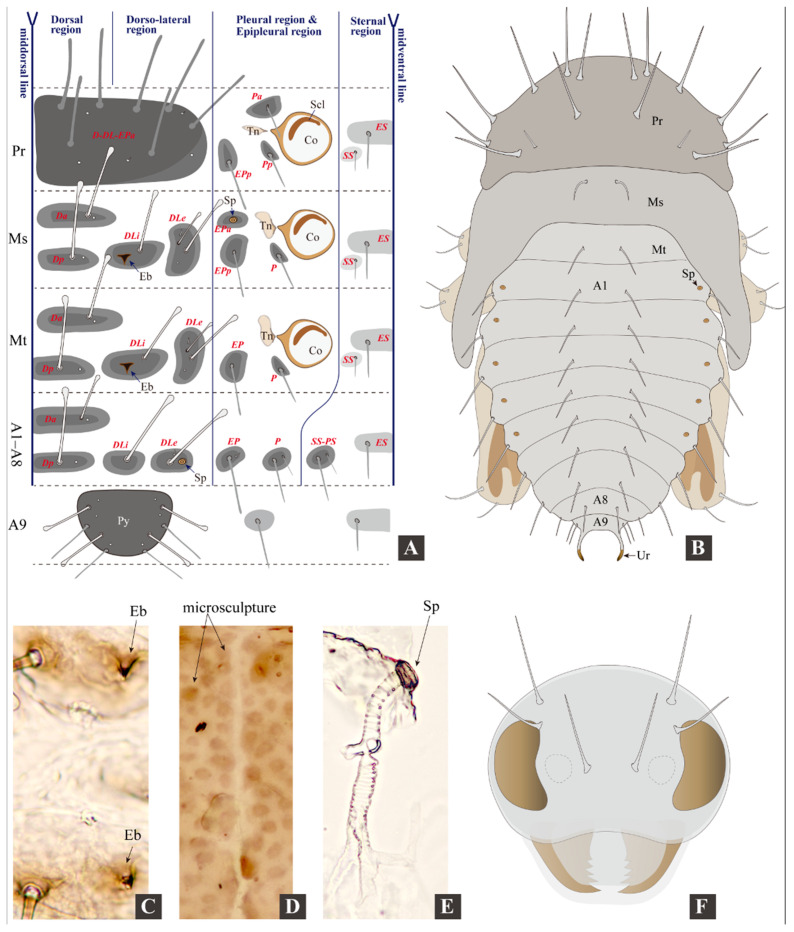
**Larval and pupal morphology of *Cangshanaltica fuanensis* sp. nov.** (**A**): Tubercular pattern of mature larva, terminology follows Kimoto (1962) [16] and Takizawa (2005) [17]. (**B**): Dorsal view of Pupa. (**C**): Egg-bursters on meso- and metathorax of larva. (**D**): Ovoid micro sculpture on pronotum of larva. (**E**): Mesothoracic spiracle. (**F**): Head of pupa. (Abbreviations of tubercles: ***D***, *dorsal*; ***DL***, *dorsolateral*; ***EP***, *epipleural*; ***ES***, *eusternal*; ***P***, *pleural*; ***PS***, *parasternal*; ***SS***, *sternellar*. Position of tubercles is denoted by lowercase letters: ***a***, *anterior*; ***p***, *posterior*; ***i***, *interior*; ***e***, *exterior. **A1***–***A9***, *abdominal segments I*–*IX*; ***Co***, *coxa*; ***Scl***, *sclerotization*; ***Eb***, *egg-burster*; ***Ms***, *mesothorax*; ***Mt**, metathorax*; ***Pr***, *prothorax*; ***Py***, *pygidium*; ***Sp***, *spiracle*; ***Tn***, *trochantin*.).

**Measurements of larvae.** Body length 0.90–2.80 mm, body width 0.30–0.80 mm. Head width 0.27–0.46 mm (Figure 3, Figure 4 and Figure 5; Table 1).

First instar: head width 0.27–0.30 mm; body length 0.90–1.50 mm, body width 0.30–0.40 mm.
Second instar: head width 0.34–0.37 mm; body length 1.50–2.30 mm, body width 0.45–0.61 mm.
Third instar: head width 0.42–0.46 mm; body length 2.00–2.80 mm, body width 0.50–0.80 mm.

Head width of second instar to that of the first instar ratio: 1.2–1.3. Head width of third instar to that of second instar ratio: 1.2–1.3.

The following descriptions are based on the third instar larvae:

**Larval morphology.** Live specimens: body eruciform; lemon yellow, semi-transparent (intestinal tract and content visible from exterior), head brown, eye spots black; legs blackish, semi-transparent; pronotum and body tubercles grey-black; ovoid micro sculpture present on body surface (only visible under high magnification, see Figure 5D). Head and pronotum with long setae (not capitate). Mesothorax, metathorax, and abdomen: dorsal and dorso-lateral region with long capitate setae, epipleural region with shorter setae (not capitate), pleural and sternal region with shorter spine-shaped setae. Spiracles present on mesothorax and abdominal segments I–VIII (Figure 3A: *Sp-ms*, *Sp-a1* to *Sp-a8*). Larvae preserved in ethanol: body “C”-shaped, pale yellow to whitish.

**Head** (Figure 4)**.** Head not retracted into prothorax, hypognathous, posterior part not ‘V’-shaped, heavily sclerotized; in frontal view globular, 1.15–1.25 times as long as wide; ovoid in lateral view. Stemmata absent, one pair of black eyespots (Figure 4A: *Eys*) present behind antenna (inside head capsule).

Epicranium (Figure 4A: *Ep*): with six pairs of long (*e1*–*e6*) and one pair of short (*e7*) setae; *e5*–*e7* situated at posterolateral part of epicranial halves. The epicranium has 12–13 pairs of sensilla (*Sen*), including four pairs of larger ones near frontal sutures. Epicranial suture (*Eps*) short, approximately half as long as endocarina (*En*).

Frons (Figure 4A: *Fr*): with three pairs of long setae (*f1*–*f3*) and one pair of sensilla; frontal sutures (*Frs*) reaching antennal sockets; median endocarina narrow ridge-shaped, extending from base of frontal sutures to clypeus.

Clypeus (Figure 4A: *Cly*): sclerotized, transverse, band-shaped, bearing 1 pair of long setae and three pairs of sensilla. Clypeus and frons divided by epistomal sulcus; epistomal ridge strongly sclerotized.

Labrum (Figure 4C,H: *Lbr*): sclerotized, transverse; bearing one pair of setae medially, one pair of setae laterally and one pair of sensilla near midline; anterior edge deeply incised (i.e., anteromedial notch), bearing numerous microtrichia; posterior edge with middle part produced posteriorly.

Mandibles (Figure 4F,J: *Md*): symmetrical, palmate, mesal membranous lobe absent (present in adults); each mandible with four sharp teeth, one sensillum near base and two setae on lateral edge, penicillus with 7–8 stiff setae, first tooth largest, remainder decrease in size.

Antenna (Figure 4E,G): weakly sclerotized, two segmented, attached to membranous area at end of frontal suture; first antennomere partly membranous, bearing one large conical sensory papilla and several sensilla; second antennomere small, bearing about four sensilla.

Maxilla (Figure 4B,D): cardo (*Ca*) small, triangular, bearing one seta, with a longitudinal band-shaped sclerotization (*Scl*); stipes (*St*) elongate with a long and curved sclerotization (*Scl*), bearing two long setae near lateral edge. Mala with galea (*Gal*) and lacinia (*Lac*) not fused; apical part of galea with approximately 5–6 setae arranged around a stout pedunculate seta (appearing two-segmented); apical part of lacinia bearing a row of peg-shaped stout setae; maxillary palpus (*Mxp*) three-segmented: first palpomere with two setae, second palpomere with two setae and one sensillum, third palpomere with one digitiform sensillum (*Dsen*).

Labium (Figure 4B,D: *Lbi*): submentum (*Smen*) trapezoid, membranous, bearing two pairs of long setae; mentum not well defined; prementum short and transparent, with prominent horseshoe-shaped mental sclerite, bearing one pair of setae at base; ligular membranous, not separated from prementum, anterior edge broadly rounded to straight, bearing numerous microtrichia; labial palpi small, two segmented; five pairs of sensilla present near labial palpi.

**Thorax.** The tubercular pattern on the right side of the thorax is illustrated in Figure 5A and described as follows (tubercle names are italicized).

Prothorax: dorsum with large *D-DL-EPa* bearing seven long setae and about five sensilla, well sclerotized. Tubercles on epipleural, pleural, and sternal regions weakly sclerotized, with poorly defined edge. Epipleural region with *EPp* bearing one long seta (not capitate). Pleural region with *Pa* and *Pp* each bearing one spine-shaped seta. Sternal region with *ES* and *SS* obsolete, each bearing one spine-shaped seta.

Mesothorax: tubercles weakly sclerotized, with poorly defined edge. Dorsal region with *Da* and *Dp* each bearing one long capitate seta and one sensillum. Dorso-lateral region with *DLi* bearing one long capitate seta and one egg-burster; *DLe* bearing two capitate setae, and two sensilla. Epipleural region with *EPa* bearing one spiracle and *EPp* bearing one long seta (not capitate). Pleural region with *P* bearing one spine-shaped seta. Sternal region with *ES* and *SS* obsolete, each bearing one spine-shaped seta.

Metathorax has the same tubercular pattern as mesothorax, except for the absence of *Epa* and spiracle; one egg-burster present on *DLi*.

**Leg** (Figure 3E)**.** One seta present on base of claw. Pulvillus present, membranous, round to ovoid, as long as tarsungulus.

**Abdomen.** Abdomen with 10 abdominal segments. The tubercular pattern on the right side of the abdomen is illustrated in Figure 5A and described as follows (tubercle names are italicized).

Segments I–VIII: with same tubecular pattern and chaetotaxy, tubercles weakly sclerotized, with poorly defined edge. Dorsal region with *Da* and *Dp* each bearing one long capitate seta and one sensillum. Dorso-lateral region with *DLi* bearing 1 long capitate seta; *DLe* bearing one long capitate seta, one setose sensillum and one spiracle. Epipleural region with *Ep* bearing one long seta (not capitate) and one setose sensillum. Pleural region with *P* bearing one long and one short spine-shaped setae. Sternal region with *SS-PS* bearing one long and one short spine-shaped setae; *ES* obsolete, bearing one spine-shaped seta.

Segment IX. Pygidium (Figure 3D) moderately sclerotized, bearing two pairs of capitate setae close to lateral edge, another two pairs of un-capitate setae on lateral edge, and about three pairs of sensilla. Lateral side with one pair of obsolete tubercles bearing one spine-shaped seta. Venter with one pair of obsolete tubercles bearing one spine-shaped seta.

Segment X not visible in dorsal view, bearing prominent eversible pygopod (Figure 3C: *Pd*).

**Remarks on larval characters.** The general appearance of *Cangshanaltica fuanensis* sp. nov. is similar to some free-living flea beetle larvae described by previous studies (see Table 1), such as *Altica* Geoffroy [20], *Ivalia* Jacoby [14], *Distigmoptera* Blake [10]. However, the larval tubercular pattern (Figure 5A) of *C. fuanensis* sp. nov. is unique among flea beetles. For instance, pleural tubercle of prothorax divided into two parts (*Pp* and *Pa*); *Epa* present on mesothorax and absent on metathorax; pygidium with four long setae; only two tubercles (*DLi* and *DLe*) present on dorso-lateral region of mesothorax, metathorax, and abdomen.

The adult morphology of *Cangshanaltica* Konstantinov et al. is close to *Ivalia* Jacoby [2]. Larval morphology of *C. fuanensis* sp. nov. also shows extreme similarity with *Ivalia korakundah* Parathapan et al., especially in the body shape, tubercular pattern, and chaetotaxy. Both larval and adult morphology suggests that they are very close lineages. Despite the resemblance in general morphological features, the larvae of *Cangshanaltica fuanensis* sp. nov. and *Ivalia korakundah* can be differentiated by the following characters: in *C. fuanensis* sp. nov. body tubercles with weak sclerotization and poorly defined edge (tubercles with moderate sclerotization and well-defined edge in *I. korakundah*), tubercle *EPa* of mesothorax absent (*EPa* of mesothorax present in *I. korakundah*), maxilla with triangular cardo (cardo narrow and transverse in *I. korakundah*).

The larval mandible of *C. fuanensis* sp. nov. is very similar to that of adults in the general palmate shape, sharp teeth, decreasing in size from the first to last tooth. The significant difference is that the adult mandible has one more tooth, and a mesal membranous lobe with dense microtrichia, which are absent in the larvae. The resemblance of their mandible shape may be related to similar feeding behavior, both are eating the leaves of the same host plant.

#### 3.2.2. Pupal Morphology

Body length 1.60–1.75 mm; width 0.90–1.10 mm; bent ventrally in lateral view. Body white–yellow to yellow–brown, bearing long, brownish, and spine-shaped setae. Head invisible from above, bearing 3 pairs of long setae. Mesothorax and abdominal segment I–V bearing one spiracle each. Pronotum bearing seven pairs of long and one pair of short setae; meso- and metanotum each bearing one pair of setae near middle. Abdominal segments I–VIII each bearing one pair of dorsal and one pair of lateral setae. Abdominal segment IX bearing two pairs of setae and one pair of distal urogomphi. Abdominal segment X invisible in dorsal view, bearing prominent eversible pygopods (Figure 5B,F).

#### 3.2.3. Egg Morphology

**Figure 6:**
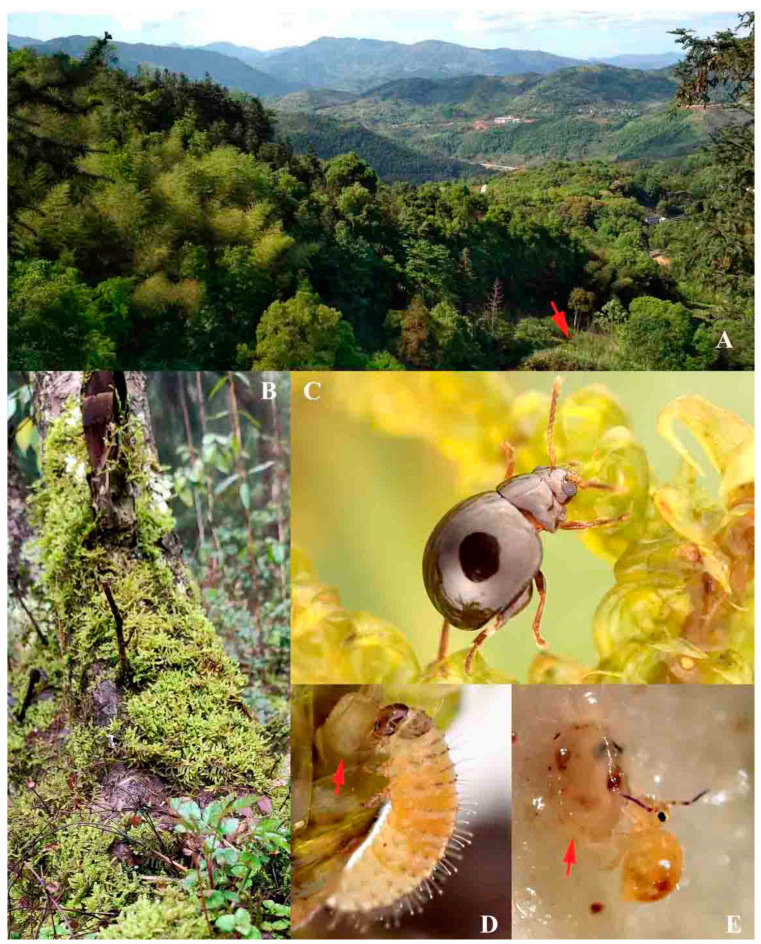
**Biology of *Cangshanaltica fuanensis* sp. nov.** (**A**): Habitat environment—a north-facing valley, arrow showing collecting spot—a deserted plum orchard with a creek flowing nearby. (**B**): Host plant *Hypnum plumaeforme* on the stem of a plum tree; they usually grow on soil, rocks, and tree stem. (**C**): An adult on the host plant. (**D**): A 2nd instar larva is eating an egg in a lab environment (cannibalism); arrow indicating an egg hidden under a moss leaf by female. (**E**): A damaged egg consumed by an unknown species of springtail (collembola) in a lab environment.

**Figure 7:**
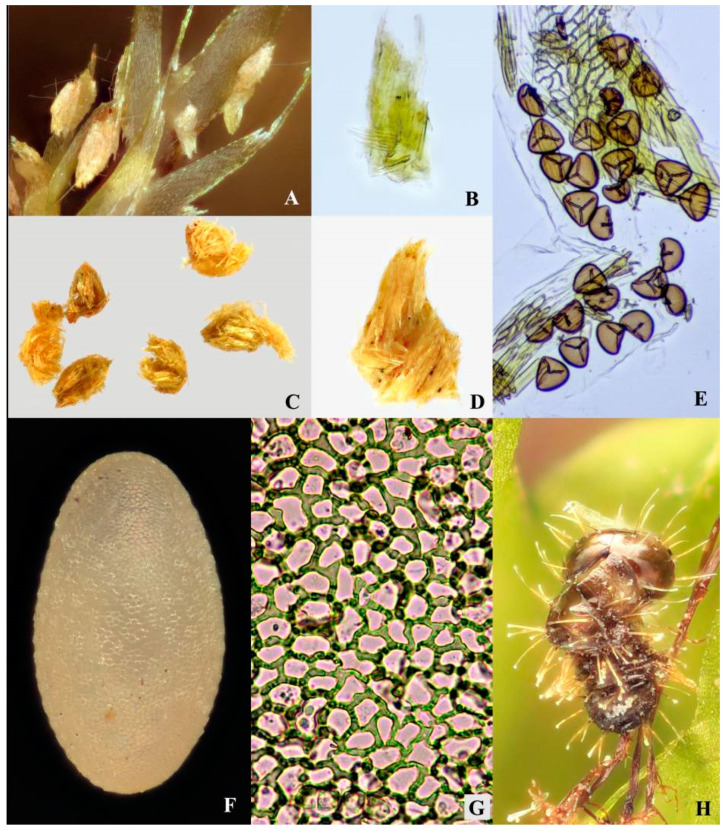
**Biology of *Cangshanaltica fuanensis* sp. nov.** (**A**,**B**): Larval feces. (**C**,**D**): Adult feces. (**E**): Contents in the intestinal tract of an adult, showing moss cells and spores. (**F**): Egg. (**G**): Micro sculpture on egg chorion. (**H**): Excuvium of larva.

**Figure 8:**
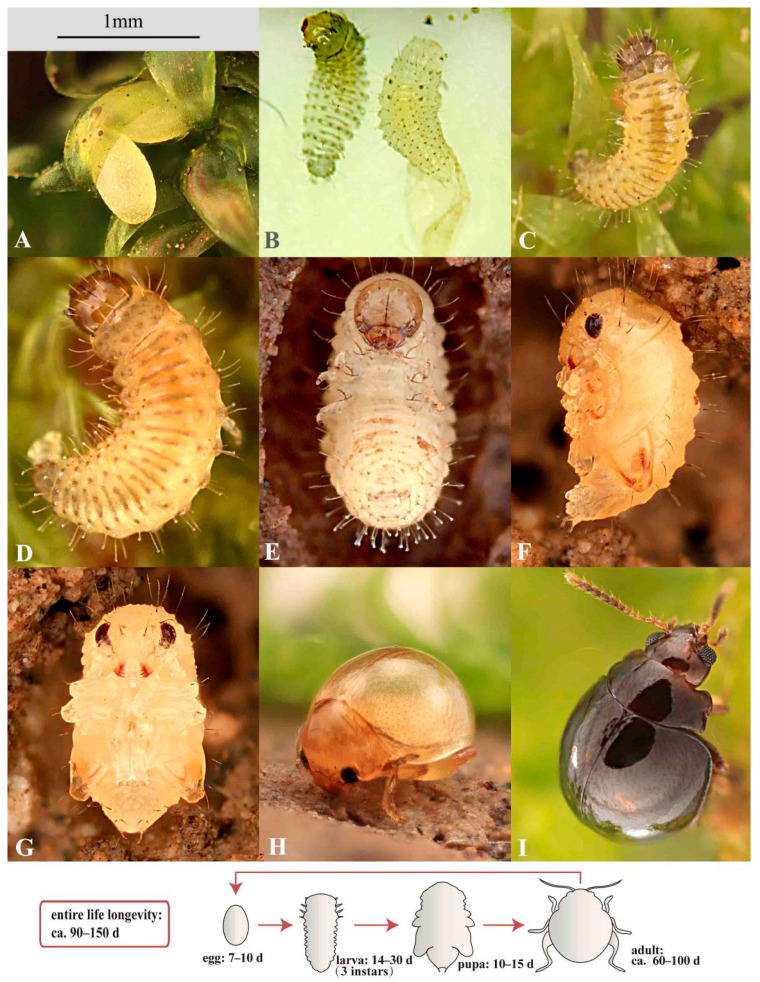
**Life cycle of *Cangshanaltica fuanensis* sp. nov.** (1 mm Scale bar is suitable for insets (**A**–**I**)). (**A**): Two eggs are laid under one moss leaf (this is a rare case; usually, there is only one egg under one leaf). (**B**): Newly hatched larvae; the individual on the right is just crawling out of the egg. (**C**): First instar larva. (**D**): Third instar larva. (**E**): Prepupal larva (with milky-white color) in pupal chamber. (**F**): Pupa, lateral view. (**G**): Pupa, ventral view. (**H**): Newly emerged adult. (**I**): Well-sclerotized adult.

Length 0.68–0.74 mm, width 0.36–0.40 mm. Egg large, egg length to female adult body length ratio: 0.4–0.5; egg width to female adult body width ratio: 0.29–0.33. Egg oval and lemon yellow. Surface with minute reticulated pattern consists of irregular polygonal cells (mostly hexagonal or pentagonal when viewed under high magnification) (Figure 6D,E, Figure 7F,G and Figure 8A; Table 2).

Remarks. The polygonal pattern on egg chorion resembles those in some other flea beetles, e.g., the polygonal pattern in *Agasicles hygrophila* Selman and Vogt (observed in current study), and the hexagonal cells further subdivided into 4 to 6 smaller ones in *Disonycha leptolineata* Blatchley [23].

### 3.3. Biology

#### 3.3.1. Life History

Approximately 30 F_0_ parent adults, 35 F_1_ eggs, 30 F_1_ larvae, 10 F_1_ pupae, and 6 F_1_ adults were observed in this study (in a lab environment, ca. 25 °C, 90–100% air humidity). The following biological data were collected based on these lab-reared individuals (Figure 8).

Life longevity lasts approximately 90–150 days. Approximately 3–4 generations per year are predicted in the type locality (Fujian, China).

**Egg stage** (Figure 7F and Figure 8A)**.** The fecundity is approximately 2–4 eggs per female. The egg stage lasts approximately 7–16 days on slightly moist to entirely wet moss. The hatching of the eggs on dry moss usually took more time than those on wet moss. It indicates that higher humidity may expedite the development of eggs.

**Larval stage** (Figure 8B–E)**.** Duration: ca. 14–30 days. Chorion ingestion is absent based on our observation. About 1–2 days before hatching, larvae are active and visible from the exterior; apex of mandibles are sclerotized and red-brown; eyespots and egg-bursters are black and prominent; larvae frequently move and rotate inside the egg chorion in this period. Before emerging, larvae contract their body and push the dilated thorax (dorsum) against egg chorion, after several times of contraction, the egg-bursters slit the chorion. Newly hatched larvae usually stay at the base of moss, they feed on both rotten and fresh leaves of the host plant. Two larvae were spotted feeding on adult feces (their feeding last ca. 2–3 min, in a lab environment). As the adult feces consist of un-digested moss leaf fragments (see Figure 7D), they are probably suitable for a food source for young larvae. It is still unknown if the feces-feeding behavior also occurs in nature.

Because the larvae are lemon yellow and semi-transparent, they are challenging to discover on the host plant (yellow-green). Furthermore, the host plant tissue in their intestinal tract is only partially digested and highly visible externally, which makes their color much closer to that of moss.

First instar. Duration: ca. 4–7 days. Head width 0.27–0.30 mm; body length 0.90–1.50 mm, body width 0.30–0.40 mm. Newly hatched individuals appear unsclerotized (Figure 8B, the individual on the right side), transparent except for red mandibles, black eyespots and egg-bursters, and light-black setae. Old individuals (Figure 8C): body becomes semi-transparent and lemon yellow; pronotum, pygidium, and tubercles light-black; mandibles brown. The number and shape of egg-bursters are similar to some other flea beetles we examined (e.g., *Agasicles hygrophila* Selman and Vogt).

Second instar. Duration: ca. 4–6 days. Head width 0.34–0.37 mm. Head width of second instar to that of first instar ratio: 1.2–1.3. Body length 1.50–2.30 mm, body width 0.45–0.61 mm. The newly molted individual with body transparent and soft. Old individual: body lemon yellow; pronotum, pygidium, and tubercles light black; mandibles reddish-brown to dark brown. Egg-bursters obsolete.

Third instar. Duration: ca. 8–13 days (including a prepupal period). Head width 0.42–0.46 mm. Head width of third instar to that of second instar ratio: 1.2–1.3. Body length 2.00–2.80 mm, body width 0.50–0.80 mm. With similar coloration as second instar. Egg-bursters obsolete.

Prepupal period (pupating period): 3–5 days, body color turned milky white. In the first 1–2 days: prepupal larvae stop feeding, crawl down to soil surface near the base of moss, excrete residual contents in the intestinal tract, and search for pupation location. Day 2–3: they build an earthen chamber, use legs to collect moist earth patches, bite off minute earth granules one by one using mouthpart, then attach them to the chamber wall; by this means, they construct the oval and smooth inner wall of the earthen chambers. Day 3–5: earthen chambers are completed, they cease movement and are not responding to minor stimuli.

**Pupal stage** (Figure 8F,G)**.** Duration: 10–15 days, abdominal segments are able to move, with eversible pygopod. In the first 1–3 days: body light-yellow; surface glabrous and transparent. Day 4–6: body yellow, semi-transparent; mandibles and compound eyes red. Day 7–8: body light yellow-brown, semi-transparent, mandibles and metafemoral springs brown, compound eyes red. Day 9–15: body yellow-brown, not transparent; eyes, legs, antennae, mouthparts and metafemoral springs well sclerotized and with brown color.

**Adult stage** (Figure 8H,I)**.** Adult longevity: ca. 60–100 days. After emergence from pupae, adults stay in the earthen cell (for approximately one day), with yellow and unsclerotized integument, and are unable to jump upon stimulation. After a few days (ca. 2–3 days), their integuments gradually turn brown, and they can perform minor jumps. After approximately 5–6 days, adults can perform explosive jumps.

#### 3.3.2. Reduction of Ovarioles and Large Egg Size

The ovaries of seven females of *Cangshanaltica fuanensis* sp. nov. were dissected in this study (Figure 2K): females have two ovaries, each ovary has only two ovarioles (Figure 2K, indicated by two arrows), which is very unusual in leaf beetles. According to [24], the ovariole number per ovary usually varies from 4 to 46 in Chrysomelidae.

Females lay significantly larger and fewer eggs than many other flea beetles (see Table 2), the maximal egg length reaches up to half of the female body length.

#### 3.3.3. Egg Hiding Behavior of Females

The females deposit one egg at a time. Eggs are laid independently and at a distance from each other. Each egg is hidden under a single leaf of the host plant (see Figure 6D, indicated by arrow). As the moss leaves are strongly convex dorso-ventrally, spoon-shaped, and slightly larger than the egg, they could well contain and cover the eggs. The eggs are not garnished with feces or litter. However, they are cryptic under moss leaves and very difficult to discover, even when viewed under the stereomicroscope. It would be more difficult to spot them when the moss is wet, and the eggs are partly immersed in water. By this means, they may be protected from potential predators (see the following text in the “Natural enemies” section). In a sporadic case, two eggs were laid adhering to each other under the same moss leaf (Figure 8A).

#### 3.3.4. Cannibalism

The cannibalism of a second instar larva on an egg was observed (see Figure 6D). The entire feeding process lasted for approximately 10 min; in the end, most of the egg contents were eaten by the larva.

#### 3.3.5. Host Plant and Feeding Habit

Both adults and larvae are polyphagous; they can feed on different host plants of different moss families. In nature, adults primarily inhabit and feed on *Hypnum plumaeforme* (Hypnaceae), which is a common moss in East Asia (including both north and south China) and grows in various habitats at different altitudes (50–4000 m above sea level) [18]. Adults also feed on *Racopilum* cf. *aristatum* (Racopilaceae) in nature. However, at the type locality, *Racopilum* cf. *aristatum* is rather scarce compared to the dominant moss species *Hypnum plumaeforme*. In a lab environment, when *Hypnum plumaeforme* and *Racopilum* cf. *aristatum* are absent, adults also casually feed on *Bazzania tridens* (Lepidoziaceae), Hylocomiaceae sp. and Thuidiaceae sp. Larvae feed on both *Hypnum plumaeforme* and *Racopilum* cf. *aristatum* in a lab environment; their feeding behavior in the natural environment was not studied.

The food source is abundant to *C. fuanensis* sp. nov. An investigation was conducted on a 30 m^2^ sample plot at the type locality in July 2020. It turned out only 15 adults were discovered on the surface of moss from 9–11 p.m. in a single night. Based on our field investigation, the host plant *Hypnum plumaeforme* is abundant, and the feeding injury on the host plant (e.g., damages on the distal end of young shoots of moss) is scarce. All these indicate the population density of this species may be low.

Adults usually feed on the distal end of moss branches, the end of young shoots are usually chopped off by their feeding, which is destructive to the host plant. In a lab environment, adults can feed on other parts of the host plant after most of the end of young shoots are eaten. Dissection of the intestinal tract of adults collected from nature shows that they also consume moss spores (Figure 7E).

At the type locality of *C. fuanensis* sp. nov., another flea beetle species-*Benedictus* sp. was discovered. In both natural and lab environment, it feeds on *H. plumaeforme*, and has very similar feeding habits, eating the distal end of young branches. The population density of *Benedictus* sp. is probably lower than *C. fuanensis* sp. nov. based on the number of specimens collected (less than 10). The competition between the two species in nature is still unknown.

Feces of larvae and adults mainly consist of undigested fragments of host plant leaf (see Figure 7B,D), which is unique in flea beetles.

#### 3.3.6. Habitat Environment

Most specimens collected in this study were extracted from moist moss in a north-facing valley with a creek flowing nearby at the type locality (Fujian, China) (Figure 6A). The surrounding mountain has north-facing steep slopes; they prevent long-time direct sunlight; therefore, they create a comparatively humid environment. There are various mosses in this area, and they are able to grow all year-round.

The adult of *Cangshanaltica fuanensis* sp. nov. is diurnal and nocturnal, they could be discovered in nature in both day and night as long as the mosses are moist. We searched for the individuals in a 30 m^2^ sample plot at the type locality in July 2020. The number of adults presented on the mosses at different times of a single day was counted: 5 adults were discovered in the early morning (6–7 a.m., mosses were slightly moist with dew); 3 adults were discovered in the late morning (10–11 a.m., mosses were almost dry); 10 individuals were discovered in the dusk (6–7 p.m., mosses were slightly moist with dew); approximately 15 adults were found in the night (9–11 p.m., mosses were wet with heavy dew). The valley we investigated was exposed to the sunlight from 9 a.m. to 5 p.m. The mosses in the open area appeared dry, and no specimens were found on them. Some individuals could be discovered on these same mosses in the night. This is because the valley became damp even in clear nights, the dew could revive the dry mosses quickly after sunset. The behavior of the larvae in nature is still unknown, as they were not found in this investigation.

Based on the laboratory observation, the arid environment is lethal for the immature stages of *C. fuanensis*. This was tested on two first instar larvae and one prepupal larva: (1) we placed the first instars in an open plastic container with 40% ± 5% air humidity, they were dead and entirely exsiccated within 2 h; (2) an earthen chamber containing a prepupal larva was taken from moist soil and placed in an open plastic container with 40% ± 5% air humidity, the prepupal larvae inside the chamber was exsiccated and killed within 8 h. Furthermore, based on our observations, the egg stage is much shorter when the moss has more moisture (see “Egg stage” section above). These observations show that the high humidity of the air, host plant, and earth is essential for the survival of immature stages.

However, an extremely wet environment (flooding environment) could also be lethal for both larvae and adults. When reared in the lab, larvae were frequently drowned dead in the minute condensed water drops on the lateral walls of rearing containers. A water immersion experiment was conducted on one adult: it was completely immersed in the distilled water, after approximately 1.5 min, it ceased movement and was not responding to stimuli.

Dormancy of *C. fuanensis* was not found at the type locality (Fujian, China). *Hypnum plumaeforme* still grows in winter in Fujian province. Numerous adults were extracted from the moss in February at the type locality (January and February are the coldest months in Fuan, Fujian prov., the lowest temperature is approximately 0 °C); these adults were still active after transferring them to a rearing container with the same temperature as the type locality. Adults of *C. fuanensis* are probably active all year round in nature, as they were collected from the field in February, May, July, August, and October. We have collected the larvae in February and October; it is unknown if they also present in other months. In a lab environment with similar air temperature as the type locality, adults and larvae could be reared throughout the year.

#### 3.3.7. Natural Enemies

Fungus gnats (Diptera: Sciaroidea sp.). In this study, one prepupal larva in the pupation chamber was attacked by two fungus gnat larvae, its first abdominal segment was ruptured by them, which led to its death. Larvae of Mycetophilidae were frequently discovered in moist moss in our study; it is still unknown if they can affect the survival of immature stages of *Cangshanaltica fuanensis* sp. nov. in nature.

Springtails. An unknown species of Sminthuroidea was observed feeding on a damaged egg of *Cangshanaltica fuanensis* sp. nov. for 5 min (Figure 6E). It is unknown if other animals already damaged the egg before the springtail’s feeding. After its feeding, only part of the chorion is left.

In this study, some other carnivorous arthropods were frequently found in the moist moss and the soil underneath it, such as rove beetle adults and larvae (Staphylinidae; usually carnivorous), adults of ground beetles (Carabidae; usually carnivorous), thrips (Thysanoptera; some groups are carnivorous), spiders and mites (Arachnida; some groups are carnivorous), centipedes (Chilopoda; usually carnivorous), etc.

#### 3.3.8. Death Feigning (Thanatosis)

When stimulated with a brush, the larvae would curl into ‘C’ shape for 30 s to 3 min before they started to move. Adults do not show prominent death feigning; instead, they can conduct several continuous jumps upon stimulation.

#### 3.3.9. Jumping Ability

Maximal jumping distance: 29 cm (approximately 200 times their body length, tested for three individuals, 60 jumps in total). Adults could perform continuous jumps upon stimulation in a lab environment. In the natural environment, when the mosses are gently disturbed, they tend to crawl away and hide; they only jump when the mosses are shaken severely, or our hands are very close to them.

### 3.4. The Modified Fan-Driven Berlese Funnel

Traditional Berlese funnel (using a light bulb as light and heating source) was frequently used for extracting the soil, leaf litter and moss inhabiting arthropods, including flea beetles [6]. These samples usually contain high level of moisture and it is very time-consuming to process these samples until they dry using traditional Berlese funnel. In this study, we developed a modified Berlese funnel using a fan and high-power PTC (positive temperature coefficient) heating elements to expedite the extracting process by removing the moisture from the samples more efficiently (see “Materials and Methods” section for more information). Both modified and traditional Berlese funnels were tested in the field. It turned out that ca. 2–4 h are required to process a slightly moist moss cushion (moss volume = 6–8 L) using the modified fan-driven Berlese funnels. In comparison, using traditional low-power Berlese funnels usually requires more than 12 h to process the same type and amount of moss sample. The modified fan-driven Berlese funnel requires ca. 4–8 h for entirely wet moss (moss volume = 6–8 L); ca. 4–5 h for moist and fluffy humus soil (volume = 4–5 L).

The modified fan-driven Berlese funnel was proven suitable for collecting moss inhabiting flea beetles. Approximately 30 adults and 15 larvae of *Cangshanaltica fuanensis* sp. nov. were successfully extracted from moss and kept alive. This may be the second most successful (based on the number of specimens) documented moss inhabiting flea beetle collection event after the collection of 83 specimens of *Kiskeya elyunque* Konstantinov and Konstantinova in Puerto Rico [1]. Some other flea beetle species inhabiting or hiding in the moss were also extracted (including *Benedictus* sp., *Chaetocnema constricta* Ruan et al., *Minota* sp.1, *Minota* sp.2, *Parathrylea septempunctata*, Jacoby, *Philopona vibex* (Erichson)). Apart from beetles, various other arthropods were also collected from moss cushions.

We tested the modified Berlese funnel for different samples: such as moist humus soil and leaf litter. In 2019, approximately 10 samples of either humus soil or leaf litter were collected under subtropical evergreen broad-leaved forest from different localities in Guangdong and Yunnan Provinces, China. Various and numerous arthropods were successfully collected.

**Figure 9:**
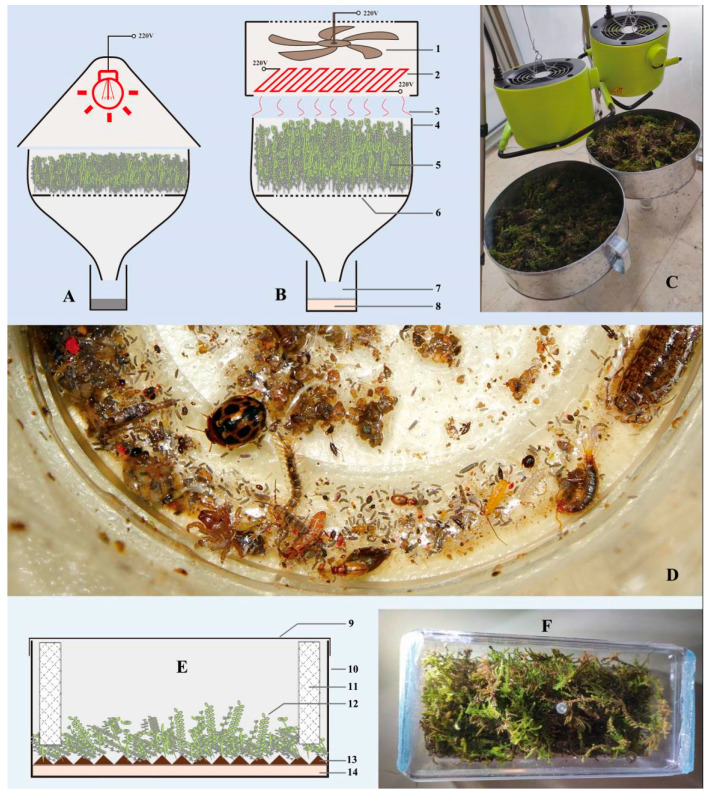
**Modified Berlese funnel and rearing methods.** (**A**). Working diagram of a typical traditional Berlese funnel. (**B**). Working diagram of the modified fan-driven high-power Berlese funnel developed in this study. (**C**). Photo of 2 sets of fan-driven high-power Berlese funnels. (**D**). Soil-inhabiting arthropods extracted using the modified Berlese funnel in a single process (cost 4–5 h, using 4–5 L of moist humus soil sample collected under subtropical evergreen broad-leaved forest, only a part of arthropods specimens is shown in this photo). (**E**). Working diagram of the container designed for rearing moss inhabiting flea beetles. (**F**). A photo of the rearing container. (**1**—*fan*; **2**—*positive temperature coefficient heating elements*; **3**—*warm airflow*; **4**—*funnel body*; **5**—*sample (moss, leaf litter or soil)*; **6**—*plastic mesh screen*; **7**—*cylindrical plastic beaker*; **8**—*a thick layer of moist paper towel to keep organisms alive (or ethanol to kill organisms)*; **9**—*lid of container*; **10**—*body of container*; **11**—*openings on container body sealed with non-woven fabrics allowing for air circulating*; **12**—*host plant*; **13**—*a shallow layer of soil*; **14**—*a layer of paper towels to maintain sufficient moisture*.)

We further conducted 10 tests using moist humus soil in Fuan, Fujian Province, then calculated arthropod specimens of each sample (volume of sample: 4–5 L), an average of ca. 400 specimens for each sample were counted. The average specimen numbers of different arthropod groups for each sample were counted as follows: Collembola ≈ 200, Arachnida ≈ 160, Insecta ≈ 30, Diplopoda ≈ 3 Chilopoda ≈ 2; Crustacea ≈ 2 (Figure 9D). It shows that this method could be useful in the studies of other soil or leaf litter inhabiting arthropods.

## 4. Discussion

### 4.1. Biology of Cangshanaltica fuanensis sp. nov.

The dense branches and leaves of bryophytes create cryptic shelters for various small-sized organisms, including moss inhabiting flea beetles. They are also an exceptional food source for flea beetles in addition to angiosperms and ferns. We found that some angiosperm feeding flea beetles also hide in the moss cushion, for example, *Chaetocnema constricta* (host plants: *Rubus* spp. and *Polygonum* sp. [28,31]), and *Philopona vibex* (host plants: *Vitex* sp. [32]).

Although the moss cushions allow for the comparatively cryptic lives of flea beetles, there were various potential natural enemies discovered in this study and in [10]. In addition to natural enemies, the cannibalism of larvae on eggs may also affect the population size of moss inhabiting flea beetles. However, due to a very low population density of larvae and adults in the field, it is unclear if cannibalism often occurs under natural conditions. Cannibalism was previously documented in many non-carnivorous insects: approximately 130 insect species and 50 species of Coleoptera [33]. Although it is unknown if the egg-eating behavior affects the survival of *C. fuanensis* sp. nov., previous studies pointed out that cannibalism may be advantageous to the individuals in the acquisition of essential nutrients, remove future competitors for food, and regulate population density [34].

It occurs that adults and larvae of *C. fuanensis* sp. nov. feed together on the leaves of *Hypnum plumaeforme*. A similar condition was previously found in *Distigmoptera borealis* [10]. Bryophytes do not have true roots; instead, they possess weak rhizoids. The flea beetle larvae seem unlikely to feed on the moss rhizoids, as they are usually hairy, senescent and weak. This may explain why larvae of *C. fuanensis* sp. nov. and *D. borealis* have the same feeding habit as adults. It is also interesting that adults of *C. fuanensis* sp. nov. and *D. borealis* are able to feed on moss spores. It is unknown if the spores could be a source of nutrients, and the cause of the spore feeding behavior remains unknown.

The reduction of ovarioles in *C. fuanensis* sp. nov. is a unique feature in leaf beetles. Suzuki ([24,35]) summarized the ovariole numbers (per ovary) for 26 species of Chrysomelidae, which vary from 4 to 46. Usually, in flea beetle species, there are 5–27 ovarioles per ovary [24]. Their studies also revealed that the ovariole number is highly variable interspecifically in Chrysomelidae. Therefore, further studies on more individuals of *C. fuanensis* sp. nov. are required to confirm our findings. A lower number of ovarioles usually indicates a corresponding limitation on the number of eggs which can be ripe at a time [36]. This partly explains why females of *C. fuanensis* sp. nov. only lay approximately 2–4 eggs. A similar condition was previously reported in some other Coleoptera. Only one ovary with a single ovariole was found in some Scarabaeinae [36,37].

Another distinct biological feature in *C. fuanensis* sp. nov. is the comparatively larger eggs. This is probably related to the reduction of ovarioles. The similar condition occurs in some other minute beetles, for instance in Ptiliidae. The mature ptiliid egg, prior to oviposition, occupies up to 1/3 of the body volume [38]; *Sphaerius* (Sphaeriusidae), and *Hydroscapha* (Hydroscaphidae) also produce a single large egg at a time [39,40]. It is unknown if the larger eggs and the reduction of ovarioles in *C. fuanensis* sp. nov. are adaptations to the unique habitat environment or are related to the small body size.

Recent studies show that some of the moss-eating leaf beetles are nocturnal and were only found on moist moss at night, for example, *Cangshanaltica sprynari* Damaška and Aston [7] and *Taiwanoshaira* spp. [12]. Based on our observation, *C. fuanensis* sp. nov. is both nocturnal and diurnal in the lab environment and nature.

### 4.2. The Modified Fan-Driven Berlese Funnel

High-power heating elements had been used in modified Berlese funnels previously (e.g., [41,42,43]). However, these modified Berlese funnels are massive and complicated. They are hard to carry and unsuitable for travel. Berlese funnels rely on electricity supply, which is not always available at the remote study sites [44]. Some alternative, electricity-free, methods include the ‘Winkler/Moczarski eclector’ [45,46]. Although this type of device is portable, light, and electricity-free, it could be time-consuming for highly moist samples such as the wet moss cushions.

In this study, we proposed a fan-driven high-power Berlese funnel for collecting moss inhabiting flea beetles. Apart from flea beetles, this method may have broader applications in collecting other organisms inhabiting soil or leaf litter. Although this method is time-saving (e.g., 2–4 h extraction period for 6–8 L slightly moist moss), it is still unknown if this could lead to incomplete sampling, which is also not known for some other extracting techniques. We have not used a light source in the modified device as it may lead to a failure of collecting some small photophobic arthropods. A recent study also shows that the heating temperature could be vital for the efficiency of extracting small animals from soil [47]. Therefore, in future applications, the optimal temperatures need to be tested for collecting different animals, and a temperature controller could be installed to adjust the heating power further.

## Figures and Tables

**Table 1:** Comparison of larval morphology between *Cangshanaltica fuanensis* sp. nov. and other flea beetles.

	*Cangshanaltica fuanensis* sp. nov.	*Ivalia korakundah*, Parathapan et al.	*Distigmoptera borealis* Blake	*Agasicles hygrophila* Selman and Vogt	*Podagricomela shirahatai* (Chûjô)	*Aphthona russica* Konstantinov et al.
Feeding type	Leaf feeding (moss)	Leaf feeding (moss)	Leaf feeding (moss)	Leaf feeding (angiosperm)	Leaf mining (angiosperm)	Root feeding (angiosperm)
Body color (mature larva)	Lemon yellow	White	Yellow	Green-black to black	Yellow	Whitish
Body shape	Eruciform; short and robust	Eruciform; short and robust	Eruciform; short and robust	Eruciform; short and robust	Slightly flattened dorso-ventrally; robust	Subcylindrical; elongate and slender
Sclerotization of tubercles	Weak, edge poorly defined	Moderate, edge well defined	Strong, edge well defined	Absent	Absent	Absent
Long capitate setae on dorsum	Present	Present	Present	Absent	Unknown	Absent
Head orientation	Hypognathous	Hypognathous	Hypognathous	Hypognathous	Prognathous	Hypognathous
Head shape	Globular	Globular	Globular	Globular	Flattened dorso-ventrally	Almost parallel-sided; slightly elongated
Posterior emargination of head	Shallow	Shallow	Moderately deep	Shallow	Strongly developed and V-shaped	Moderately deep
Stemmata	Absent	Absent	Absent	1 pair	Absent	Absent
Eyespot	Present	Present (based on image)	Unknown	Absent	Present	Unknown
Epicranial suture	Short	Short	Short	Short	Absent	Short
Shape of Endocarina	Narrow ridge	Unknown	Narrow ridge	Narrow ridge	Strongly developed Median bulge	Narrow ridge
Anterior edge of labrum	Deeply concave	Deeply concave	Moderately concave	Slightly concave at middle	Convex	Slightly concave
Number and shape of mandibular teeth	4; sharp, with similar size	4; largest tooth bearing minute serration	3 sharp and 1 blunt	3 large and 1 small	4; all blunt	4 sharp long and 1 small

Source of data: *Cangshanaltica fuanensis* sp. nov.—current study; *Ivalia korakundah* [14]; *Distigmoptera borealis* Blake [10]; *Agasicles hygrophila*—observed in the current study; *Podagricomela shirahatai* [21]; *Aphthona russica* [22].

**Table 2:** Larger egg size and smaller egg number in *Cangshanaltica fuanensis* sp. nov. compared with several other flea beetles.

	Egg Length and Width	Adult Body Length and Width	Egg Length to Adult Body Length Ratio	Egg Width to Adult Body Width Ratio	Egg Numbers Laid by Female
*Cangshanaltica fuanensis* sp. nov.	0.68–0.74 mm; 0.36–0.40 mm	1.5–1.7 mm; 1.2–1.3 mm (female)	0.40–0.50	0.28–0.33	Ca. 2–4, laid separately
*Agasicles hygrophila* Selman and Vogt	1.26 mm; 0.54 mm	6 mm; 3 mm	0.21	0.18	>50, laid in clusters
*Altica caerulescens* (Baly)	0.85 mm; 0.35 mm	4 mm; 2 mm	0.21	0.18	Unknown
*Altica fragariae* Nakane	0.84 mm; 0.37 mm	3.8 mm; 2 mm	0.22	0.19	>100, laid in clusters
*Chaetocnema ingenua* Baly	0.75 mm; 0.35 mm	1.9–3 mm; 0.9–1.57 mm	0.25–0.39	0.22–0.39	Ca. 100, laid in clusters
*Disonycha leptolineata* Blatchley	1.77–2.23 mm; 0.66–1.09 mm	6.2–7.5 mm; 3.4–4.5 mm	0.24–0.36	0.15–0.32	Unknown, laid in clusters
*Phyllotreta striolata* (Fabricius)	0.37 mm; 0.21 mm	1.8–2.4 mm; 0.9 mm	0.15–0.21	0.23	>25, laid in clusters

Source of data for egg length and adult body length: *Agasicles hygrophila*—measured in current study; *Altica* spp. [25]; *Chaetocnema ingenua* Baly [26,27,28]; *Disonycha leptolineata* Blatchley [23]; *Phyllotreta striolata* [29,30].
